# Similar changes in neuropsychological functioning in english and spanish speaking HIV patients

**DOI:** 10.1002/brb3.1083

**Published:** 2018-07-25

**Authors:** Onoja Akpa, Sachiko Miyahara, Babafemi Taiwo, Scott Evans, Baiba Berzins, Kevin Robertson

**Affiliations:** ^1^ Department of Epidemiology and Medical Statistics College of Medicine University of Ibadan Ibadan Nigeria; ^2^ Harvard T.H. Chan School of Public Health Boston Massachusetts; ^3^ Division of Infectious Diseases Northwestern University Feinberg School of Medicine Chicago Illinois; ^4^ Center for Global Health Northwestern University Chicago Illinois; ^5^ Departments of Neurology University of North Carolina at Chapel Hill Chapel Hill North Carolina

**Keywords:** antiretroviral therapy, HIV/AIDS infection, neuropsychological functioning, primary language

## Abstract

**Objective:**

Primary language has been reported to influence the results of neuropsychological (NP) testing. We sought to determine whether being a primary Spanish versus English speaker affects changes in neuropsychological evaluations in persons living with HIV.

**Method:**

Data from 209 (188 English speakers and 21 Spanish speakers) ART‐naïve HIV‐infected adults were extracted from ACTG A5303, a 48‐week randomized clinical trial of two HIV treatment regimens. Participants’ mean (standard deviation) age and years of education were 35.1 (10.7) and 14.3 (2.7) years respectively. Changes from baseline to week 48 of antiretroviral therapy (ART) in individual, total, and domain *z*‐scores for NP tests and Global Deficit Scores (GDS) were compared between the primary languages using linear regression models, adjusted for baseline scores and years of education.

**Results:**

Baseline demographic characteristics were comparable except Spanish speakers had less years of education than the English speakers (*p* < 0.001). Although differences in some NP measures and domains were detected at baseline, the adjusted changes in individual, total and domain NP *z*‐scores from baseline to 48 weeks of ART were not significantly different between the two primary language groups**.** The 48‐week changes in GDS were also similar.

**Conclusion:**

Changes in NP during ART were similar between English and Spanish speaking HIV‐infected individuals for all NP measures. This suggests that studies of longitudinal changes in NP can pool participants across these languages.

## 1 INTRODUCTION

Although combination antiretroviral therapy (ART) is associated with improvements in neurocognition among persons living with HIV, many suffer from a spectrum of neuropsychological (NP) impairments known collectively as HIV‐associated neurocognitive disorders (HAND) despite ART (Antinori et al., 2007; Heaton, Clifford, Franklin, Woods, & Ake, 2010; Heaton, Franklin, Ellis, McCutchan, & Lentendre, 2011). HAND can manifest as difficulty with executive functioning, information processing speed, motor deficits, and/or memory deficits (Smith et al., 2014). Accordingly, neuropsychological batteries used for HAND diagnosis must be comprehensive in order to adequately assess several neurocognitive domains (Antinori et al., 2007).

Primary or native language is one of the factors reported to affect the results of neurocognitive testing in a longitudinal study (Blake, Ott, Villanyi, Kazhuro, & Schatz, 2015). This impact is multifactorial, and not due to a sole difference in native language. Primary language may influence NP testing through linguistic and cultural factors, which may systematically influence test administration, participant performance, or operator interpretation. In the US, Hispanics constitute a significant proportion of the population of persons living with HIV (Mindt et al., 2003); approximately 20% of people living with HIV infection in 2011 (Center for Disease Control and Prevention, 2015a), and 23% of new HIV infections in 2013 (Arya et al., 2013; Center for Disease Control and Prevention, 2015b). Small studies limited to Spanish speakers with HIV in the United States have shown that instruments such as the HIV Dementia Scale may be appropriate for NP screening in this population, while other instruments such as the International HIV Dementia Scale may not perform as well (Levine et al., 2011; López et al., 2016; Mindt et al., 2003). Instruments such as HIV/University of Miami Annotated Neuropsychological test battery in Spanish (HUMANS) have been developed for use in Spanish and English speakers, but these need validations in large comparative studies of Spanish versus English speakers (Wilkie et al., 2004). Overall, a better understanding of how primary or native language affects NP testing and results of clinical trials would be critical to the care of culturally and linguistically divergent individuals with HIV infection in the United States.

Our aim in this study was to determine whether being a primary Spanish versus English Speaker had an effect on NP outcomes reported in AIDS Clinical Trials Group (ACTG) study A5303, a randomized controlled trial where ART naïve persons living with HIV experienced improvements in NP performance during ART with no significant differences between two regimens evaluated.

## 2 METHODS

A5303 was a phase 2, prospective, double‐blind, placebo controlled, multicenter, 48‐week study of maraviroc 150 mg or tenofovir disoproxil fumarate (tenofovir) 300 mg, each plus darunavir/ritonavir 800/100 mg and emtricitabine 200 mg in participants infected with the R5‐tropic HIV‐1 (Taiwo et al., 2015). Participants were ART naïve adults (aged ≥18 years) recruited from 33 AIDS Clinical Trials Group (ACTG) and four Adolescent Trials Network study sites in the USA. Participants in A5303 were randomized in a 1:1 ratio and stratified by plasma HIV‐1 RNA < or ≥100,000 copies/ml and age <30 or ≥30 years (Taiwo et al., 2015).

All study participants underwent NP assessment before ART initiation (week 0), and at week 48 (defined as week 44–54) of ART. Eleven NP tests were used to assess six domains: fine motor (Grooved pegboard dominant, Grooved pegboard non‐dominant), speed of processing (Digit symbol, Trail making A), executive functioning (Trail making B, Letter fluency FAS, Semantic verbal fluency), verbal learning (HVLT‐R Learning trials), verbal memory (HVLT‐R Delayed recall, HVLT‐R Recognition), and attention (WAIS‐III symbol search). The NP tests that differed for Spanish‐speaking participants were: HVLT (different words), Letter Fluency (different letters), Stroop (color names), and Woodcock Munoz (instead of WRAT‐4). Spanish norms were used for the Woodcock Munoz (manual), Stroop (Mitrushina, 2005), Letter and Category Fluency (Mitrushina, 2005). US English norms were used for the HVLT‐R (manual), Trailmaking A and B (Heaton, Miller, Taylor, & Grant, 2004), Grooved Pegboard (Heaton et al., 2004), WAIS III–Digit Symbol (Heaton et al., 2004) and WAIS‐III Symbol Search (WAIS III manual) (Heaton et al., 2004). The NP instructions for Spanish‐speaking participants were administered by Spanish speaking staff, each of whom received appropriate training and certification under the supervision of a neuropsychologist (Kevin Robertson). Staff training was provided through in‐person training at the annual ACTG meetings, video training films, and PowerPoint presentations. After the initial training, subsequent review of the training materials and recertification of the research staff occurred at least annually.

The present analysis (*N* = 209) was restricted to the 188 primary English speakers and the 21 primary Spanish speakers who remained on their randomized maraviroc or tenofovir containing regimen through week 48, and had NP data available at both baseline and week 48. Primary language of the study participants in the parent study was defined by self‐report. Eighteen participants whose primary language was unknown were excluded from this analysis.

Baseline characteristics were compared between language groups using Wilcoxon test (for continuous data) and Chi‐square test (for categorical data). All individual NP scores were standardized by age, gender, race, and years of education to create *z*‐scores using comparison normative data from the best available sources (Heaton et al., 2004; Mitrushina, 2005; Strauss, 2006). The change from baseline to week 48 of ART (48‐week change) in individual NP *z*‐score was computed for each participant as the *z*‐score at week 48 minus the score at baseline. Domain *z*‐scores were computed as the average of standardized individual NP *z*‐scores in the respective domains while total *z*‐score was computed as the average of the individual NP *z*‐scores. We also computed deficit scores, which remove the ‘sum to zero’ effect when adding positive and negative performances to create composite scores, and emphasize impairment over average or better performances. Individual Deficit Scores (DS) were derived from the standardized *z*‐scores as follows: DS = 0 (normal) if *z*‐score >−1.0, DS = 1 (mild to normal) if *z*‐score is [>−1.5, ≤−1.0], DS = 2 (mild) if *z*‐score is [>−2.0, ≤−1.5), DS = 3 (moderate) if *z*‐score is [>−2.5, ≤−2.0), DS = 4 (moderate to severe) if *z*‐score is [>−3.0, ≤−2.5), and DS = 5 (severe) if *z*‐score ≤−3.0. The domain DS was calculated as the average of the test DS comprising the domain, while the Global Deficit Score (GDS) was computed as the average of the 15 individual DSs (Blackstone et al., 2012; Carey et al., 2004). The 48‐week changes in NP *z*‐scores were compared between the 188 English and the 21 Spanish speakers regardless of treatment arms since there were no differences in NP *z*‐scores between the A5303 treatment arms.

The individual, total, GDS and domain *z*‐scores for the NP test between the two language groups were compared using linear regression models, adjusting for years of education. The 48‐week changes in these scores (week 48 minus baseline) between the groups were also compared using regression models. Since some of the baseline *z*‐scores and years of education (treated as continuous) were significantly different between the groups, they were included in the models as covariates. For example, to compare the 48‐week changes in digit symbol *z*‐score between the groups, the baseline digit symbol *z*‐score and the years in education were treated as covariates in the model. The Benjamini‐Hochberg method (Benjamini & Hochberg, 1995) was applied to adjust *p*‐values for multiple comparisons.

## 3 RESULTS

Baseline characteristics of the 209 participants are shown in Table 1. The mean (*SD*) age was 35.1 years (10.7), 91% were male, 6% were either currently or previously intravenous drug users, 90% were English speakers, 10% were Spanish speakers, and 71% had at least some college education. The mean (*SD*) CD4 count and plasma HIV‐1 RNA level were 418 cells/mm^3^ (205.8) and 4.5 log_10_ copies/ml (0.6) respectively. The demographic characteristics were generally balanced between the primary languages, except Spanish speakers had less years of education compared to the English speakers (*p* = 0.025).

**Table 1:** Baseline characteristics

Participants’ demographics	Primary language		Total (*N* = 209)	*p*
	English (*N* = 188)	Spanish (*N* = 21)		
Age (years), Mean ± *SD*	35.2 (10.9)	34.4 (8.5)	35.1 (10.7)	0.973*
Gender				
Male	172 (91%)	19 (90%)	191 (91%)	0.875**
Female	16 (9%)	2 (10%)	18 (9%)	
Race/ethnicity				
White Non‐Hispanic	98 (52%)	0 (0%)	98 (47%)	<0.001**
Black Non‐Hispanic	65 (35%)	0 (0%)	65 (31%)	
Hispanic (regardless of race)	20 (11%)	21 (100%)	41 (20%)	
Asian, Pacific Islander	2 (1%)	0 (0%)	2 (1%)	
American Indian, Alaskan native	1 (1%)	0 (0%)	1 (0%)	
More than one race	2 (1%)	0 (0%)	2 (1%)	
Intravenous drug history				
Never	175 (93%)	21 (100%)	196 (94%)	0.461**
Currently	1 (1%)	0 (0%)	1 (0%)	
Previously	12 (6%)	0 (0%)	12 (6%)	
Education (years)				
Mean ± *SD*	14.5 ± 2.5	12.3 ± 3.6	14.3 ± 2.7	0.025*
Less than HS graduate (<12 years)	11 (6%)	7 (33%)	18 (9%)	<0.001**
HS graduate with no college (12 years)	41 (22%)	1 (5%)	42 (20%)	
Some college < Bachelor dg (12 < −15 years)	64 (34%)	7 (33%)	71 (34%)	
Bachelor degree w/no post‐grad (16 years)	42 (23%)	6 (29%)	48 (23%)	
Postgraduate education (>16 years)	28 (15%)	0 (0%)	28 (14%)	
Unknown	2	0	2	
CD4 counts (cells/mm^3^), Mean (*SD*)	423.4 (208.6)	372.0 (176.6)	418.2 (205.8)	0.218*
HIV‐1 RNA (log10 copies/ml), Mean (*SD*)	4.5 (0.6)	4.4 (0.7)	4.5 (0.6)	0.568*

Notes. *SD*: standard deviation.

*Wilcoxon test was used. **Chi‐square test was used.

Results of NP testing at baseline and the 48‐week changes in *z* score by primary language are shown in Table 2. At baseline, after adjusting for education level, English and Spanish speakers showed significant differences in four (Digit symbol, HVLT‐R recognition, Letter Fluency, and Symbol Search) of the eleven NP measures. There were also significant baseline differences in the total GDS score and in one (Speed of Processing) of the six NP domains evaluated.

**Table 2:** Baseline and 48‐week changes in *z*‐score by primary language

	Baseline neuropsychological performance				48‐week change in neuropsychological performance			
	Primary language		Total (*N* = 209)		Primary language		Total (*N* = 209)	
	English (*N* = 188)	Spanish (*N* = 21)			English (*N* = 188)	Spanish (*N* = 21)		
	Median (IQR)	Median (IQR)	Median (IQR)	*p* *	Median (IQR)	Median (IQR)	Median (IQR)	*p* **
Neuropsychological measures								
Digit symbol *z*‐score	0.33 (−0.67, 1.00)	−1.33 (−2.00, −0.67)	0.00 (−0.67, 1.00)	<0.001	0.33 (−0.33, 0.67)	0.33 (0.00, 0.33)	0.33 (−0.33, 0.67)	0.993
Grooved pegboard dominant *z*‐score	−0.40 (−1.00, 0.30)	0.20 (−0.70, 0.70)	−0.40 (−1.00, 0.40)	0.191	0.40 (0.00, 1.30)	0.40 (−0.10, 1.20)	0.40 (0.00, 1.30)	0.993
Gooved pegboard nondominant *z*‐score	−0.65 (−1.10, 0.20)	0.00 (−0.70, 0.60)	−0.60 (−1.10, 0.20)	0.041	0.40 (0.00, 0.80)	0.40 (−0.40, 1.10)	0.40 (0.00, 0.90)	0.993
HVLT‐R learning trials *z*‐score	−0.40 (−1.20, 0.40)	−1.20 (−1.70, −0.20)	−0.50 (−1.30, 0.30)	0.016	0.40 (−0.20, 1.00)	0.50 (−0.50, 1.00)	0.40 (−0.20, 1.00)	0.993
HVLT‐R delayed recall *z*‐score	−0.10 (−1.00, 0.90)	−0.30 (−1.20, 0.40)	−0.10 (−1.00, 0.90)	0.343	0.00 (−0.30, 0.90)	0.25 (−0.60, 0.90)	0.00 (−0.30, 0.90)	0.993
HVLT‐R recognition *z*‐score	0.70 (−0.10, 0.80)	−0.60 (−1.85, 0.25)	0.70 (−0.60, 0.80)	<0.001	0.00 (−0.30, 0.40)	−0.10 (−0.55, 0.35)	0.00 (−0.30, 0.40)	0.620
Semantic verbal fluency *z*‐score	−0.10 (−0.70, 0.40)	0.10 (−0.50, 0.90)	−0.10 (−0.70, 0.40)	0.501	0.00 (−0.45, 0.80)	−0.40 (−0.80, 0.40)	0.00 (−0.50, 0.80)	0.400
Letter fluency *z*‐score	−0.40 (−0.90, 0.40)	−1.00 (−1.80, 0.00)	−0.40 (−1.00, 0.30)	0.009	0.40 (0.00, 1.10)	0.70 (0.00, 1.10)	0.40 (0.00, 1.10)	0.993
Trail making A *z*‐score	−0.30 (−1.00, 0.35)	−0.70 (−1.40, 0.00)	−0.30 (−1.10, 0.30)	0.119	0.50 (0.00, 1.30)	0.60 (−0.20, 1.20)	0.50 (0.00, 1.30)	0.993
Trail making B *z*‐score	0.10 (−0.70, 0.90)	−0.60 (−0.80, 0.35)	0.00 (−0.75, 0.90)	0.070	0.45 (0.00, 0.90)	0.40 (0.00, 0.50)	0.40 (0.00, 0.90)	0.340
Symbol search *z*‐score	0.33 (−0.33, 1.00)	−1.33 (−2.33, 0.33)	0.33 (−0.33, 1.00)	0.002	0.33 (0.00, 0.67)	0.33 (0.00, 1.00)	0.33 (0.00, 0.67)	0.993
Total *z*‐score	−0.13 (−0.51, 0.28)	−0.51 (−0.93, −0.28)	−0.17 (−0.56, 0.25)	0.005	0.29 (0.08, 0.51)	0.33 (0.15, 0.52)	0.29 (0.09, 0.51)	0.993
Total GDS *z*‐score	0.27 (0.07, 0.62)	0.73 (0.36, 1.00)	0.33 (0.09, 0.67)	0.001	−0.07 (−0.27, 0.00)	−0.17 (−0.55, 0.00)	−0.09 (−0.27, 0.00)	0.993
Domains of neuropsychological performance								
Fine motor *z*‐score	−0.50 (−1.00, 0.10)	−0.10 (−0.70, 0.40)	−0.45 (−1.00, 0.20)	0.084	0.40 (0.00, 0.80)	0.20 (0.00, 1.00)	0.40 (0.00, 0.80)	0.993
Speed of processing *z*‐score	−0.08 (−0.63, 0.55)	−0.85 (−1.45, −0.32)	−0.15 (−0.71, 0.51)	<0.001	0.30 (−0.11, 0.73)	0.37 (0.00, 0.77)	0.31 (−0.09, 0.73)	0.340
Executive functioning *z*‐score	−0.06 (−0.55, 0.38)	−0.70 (−0.93, −0.03)	−0.10 (−0.60, 0.36)	0.024	0.34 (0.00, 0.66)	0.23 (−0.27, 0.50)	0.34 (−0.03, 0.63)	0.993
Verbal learning *z*‐score	−0.40 (−1.20, 0.40)	−1.20 (−1.70, −0.20)	−0.50 (−1.30, 0.30)	0.016	0.40 (−0.20, 1.00)	0.50 (−0.50, 1.00)	0.40 (−0.20, 1.00)	0.340
Verbal memory *z*‐score	0.10 (−0.60, 0.80)	−0.45 (−1.15, −0.10)	0.00 (−0.70, 0.80)	0.023	0.10 (−0.15, 0.65)	0.05 (−1.00, 0.90)	0.10 (−0.20, 0.65)	0.993
Attention *z*‐score	0.02 (−0.74, 0.51)	−1.33 (−2.33, 0.33)	0.00 (−0.87, 0.50)	0.029	0.17 (−0.21, 0.59)	0.33 (0.00, 0.67)	0.18 (−0.15, 0.59)	0.993

Notes. GDS: Global Deficit Score; HVLT‐R: Hopkins Verbal Learning Test‐Revised; IQR: interquartile range.

*p‐Values are from linear regression models adjusted for education level at baseline and are adjusted by the Benjamini‐Hochberg method. **p‐Values are originally from linear regression models adjusted for baseline *z*‐scores and education level, and are adjusted by the Benjamini‐Hochberg method.

The 48‐week changes in NP performance were adjusted for both baseline *z*‐scores and education level since some baseline scores remained significantly different even after adjusting for education level. No significant differences in 48‐week changes were detected between English and Spanish speakers in individual, total and domain NP *z*‐scores. The 48‐week changes in GDS were similar as well; −0.07 (−0.27, 0.00) for English speakers and −0.17 (−0.55, 0.00) for Spanish speakers (*p* = 0.993). Since there were no longitudinal differences between the groups, it is not unlikely that there were no significant differences in practice or learning effect between the two groups.

The Stroop *z*‐scores and the WRAT‐4 were not compared because some sites chose not to administer the tests to Spanish speakers and approximately 65% of the Spanish speakers had missing scores. However, missing Stroop and WRAT‐4 tests for the Spanish speakers did not affect the *z*‐score and GDS comparisons as they were based on the available NP scores.

## 4 DISCUSSION

We compared NP outcomes between Spanish and English Speakers in ACTG study A5303, a randomized, placebo controlled trial that employed validated NP instruments. After adjusting for differences in baseline scores and education level, 48‐week median changes in NP were similar between English and Spanish speakers. Importantly, the total *z*‐score and the GDS, which are commonly used composite measures of NP, underwent comparable longitudinal changes in the two language groups. These results suggest that although English and Spanish speakers may demonstrate some differences in NP performance at a single time point, studies of longitudinal NP changes in these populations can pool participants across languages.

It is notable that following adjustments for multiple comparisons, all NP measures and associated domains demonstrated similar longitudinal changes in English and Spanish speakers over the 48‐week study period. Primary language has been reported to influence the results of NP testing (Boonea, Victor, Wen, Razani, & Pont'on, 2007; Deák, 2014). However, other studies, and the absence of significant differences in change over 48 weeks in the current study, indicate that this is not necessarily the case, perhaps when language and cultural differences are accounted for, both in the content and administration of the relevant tests (Federman, Cole, & Sano, 2009; Lopez & Taussig, 1991). The differences at baseline between the language groups, on the other hand, may partly reflect linguistic and cultural factors that are unconnected to years of education. A challenge for investigators is it may be difficult to control for all language and cultural variations for some specific test batteries. For instance, virtually all widely used standard instructions for NP tests were originally created in the English language, and some aspects may not be easily translated verbatim and so, accuracy of contents and ease of understanding may be degraded during translation from one language to another. Even when translation is verbatim, variations in cultural contexts and nuanced interpretations of the same concept across languages may affect the respondent's answer (Federman et al., 2009) and skew the results of neuropsychological evaluations. While we addressed this by administering NP tests in Spanish to Spanish speakers, there might have been subtle residual language and culture dependent factors. Other studies, though in the general population from different cultures and ethnic groups, found differences in cognitive performances on a number of standardized tests (including tests of learning efficiency, IQ tests, and problem solving; Federman et al., 2009; Lopez & Taussig, 1991; Robertson, Liner, & Heaton, 2009). It is uncertain whether unmeasured cultural or language differences also contributed to their results. Conversely, we cannot exclude the possibility that some of the baseline differences observed in our study are due to meaningful differences in NP performance that are independent of unmeasured cultural or linguistic factors.

Nevertheless, the importance of defining culturally sensitive psychological assessment cannot be over emphasized (Boonea et al., 2007; Deák, 2014; Federman et al., 2009; Lopez & Taussig, 1991; Robertson et al., 2009). NP testing instruments that are heavily language laden may be expected to be more susceptible to language and cultural differences though such susceptibility was not apparent between the Spanish and English speakers in the current study. On the other hand, instruments that are less language laden such as fine and gross motor assessments, including timed gait, grooved pegboard, and finger tapping would be expected to be more resilient (Boonea et al., 2007; Deák, 2014; Federman et al., 2009; Lopez & Taussig, 1991) but it is noted that non‐verbal measures are also affected by language and culture (Barac & Bialystok, 2012; Rosselli & Ardila, 2003).Although our study was not designed to specifically investigate this question, the results suggest that a range of tests can be used successfully when evaluating longitudinal changes.

One of the strengths of our study is that baseline CD4 cell counts were similar between the groups, a variable which is known to affect NP outcomes in individuals infected with HIV. Further at baseline, English primary speakers had more years of education than the Spanish primary speakers; however, this educational disparity is consistent with the demographic characteristics of the United States. We corrected for baseline educational differences when determining the baseline NP test scores and also in regression analysis of changes in NP at week 48. Some differences at baseline between the language groups were not attributable to differences in educational level. We corrected for these as well in our analysis of the 48 week changes by adjusting for baseline *z* scores. Our study involved English and Spanish speakers only, and may have minimal power due to the small sample size for the Spanish speakers, hence the results may not be generalizable to other language pairings. Moreover, we did not collect detailed information on the language and educational backgrounds of the Spanish participants (e.g., whether they were born and educated in the US or a Spanish‐speaking country, the age at which they came to the United States if born elsewhere, and their degree of bilingualism). Since these variables may impact NP testing results, our results may not be generalizable to all Spanish speakers. Also, our secondary conclusion that Spanish‐speakers performed less well on specific NP tests may be complicated by the cross‐sectional baseline data and the nonequivalent normed *z*‐scores used for the two primary languages included in analysis.

In conclusion, the results of this study suggest that comparative NP assessments can be conducted in English‐ and Spanish‐speaking persons living with HIV; specifically, that studies of longitudinal changes in NP may pool participants across these languages, provided culturally sensitive methodology is applied. Studies with a larger population of Spanish speakers and longer follow‐up are needed to validate our results.

## CONFLICT OF INTEREST

There authors have no conflict of interest to declare.
